# Family adjustment to COVID‐19 lockdown in Italy: Parental stress, coparenting, and child externalizing behavior

**DOI:** 10.1111/famp.12686

**Published:** 2021-07-01

**Authors:** Michele Giannotti, Noemi Mazzoni, Arianna Bentenuto, Paola Venuti, Simona de Falco

**Affiliations:** ^1^ Department of Psychology and Cognitive Sciences Observation, Diagnosis and Education Lab University of Trento Rovereto, Trento Italy

**Keywords:** COVID‐19, Parents, Children, Parental stress, Externalizing behaviors, Coparenting

## Abstract

Evidence of psychological distress in families during COVID‐19 outbreak are arising. However, the perceived changes in psychological adjustment during home confinement with respect to the period before the pandemic have not been addressed yet. Moreover, little is known about the role of coparenting and specific COVID‐19 contextual variables on parental stress and children's behavioral difficulties in the Italian context. Using a cross‐sectional survey, we collected data on 841 Italian parents of children aged 3–11 years with typical development during the home confinement (20th April–18th May). We analyzed levels of parental stress, coparenting, and child externalizing behaviors before and during the home confinement. Additionally, hierarchical regressions were performed to investigate predictors of parental stress and child externalizing behaviors during the lockdown. Results showed that parental stress (especially in mothers) and child externalizing behaviors increased during the lockdown period. Coparenting was a strong predictor of parental stress, together with being a mother, younger child age, less time dedicated to the child, and scarce feasibility of remote working. Besides, child externalizing behaviors were predicted by male gender, less parental time dedicated to the child, higher parental stress, and child distance learning workload. Our findings indicate a negative impact of COVID‐19 lockdown in both parents and children, suggesting that positive coparenting and time dedicated to children may help to reduce the detrimental effect of pandemic restrictions on family adjustment.

## Resumen

Están surgiendo datos sobre el distrés psicológico en las familias durante el brote de la COVID‐19. Sin embargo, aún no se han abordado los cambios percibidos en la adaptación psicológica durante el confinamiento. Este estudio tuvo como finalidad investigar los cambios en los niveles de estrés de los padres, las dificultades conductuales de los niños y la cocrianza antes y durante el confinamiento en el hogar. Además, analizamos los factores pronósticos de estrés por la crianza y las conductas de exteriorización de los niños durante el confinamiento. Utilizando una encuesta representativa, recogimos datos sobre 841 padres italianos durante el confinamiento en el hogar (desde el 20 de abril hasta el 18 de mayo). Analizamos los niveles de estrés de los padres, la cocrianza y las conductas de exteriorización de los niños antes y durante el confinamiento. Se realizaron regresiones jerárquicas para investigar los factores pronósticos de estrés de los padres y las conductas de exteriorización de los niños durante el confinamiento. El estrés de los padres (especialmente de las madres) y las conductas de exteriorización de los niños aumentaron durante el periodo de confinamiento. La cocrianza se mantuvo estable y fue un factor pronóstico fuerte del estrés de los padres, junto con ser madre, niños más pequeños, menos tiempo dedicado al niño y escasa viabilidad de trabajar virtualmente. Las conductas de exteriorización de los niños fueron pronosticadas por el género masculino, menos tiempo de los padres dedicado al niño, un mayor estrés de los padres y la cantidad de actividades de la enseñanza a distancia del niño. Nuestros resultados indican un efecto negativo del confinamiento por la COVID‐19 tanto en los padres como en los niños e indican que la cocrianza positiva y el tiempo dedicado a los niños pueden contribuir a reducir el efecto perjudicial de las restricciones de la pandemia en la adaptación familiar.

## 摘要

有证据表明，新冠肺炎疫情期间有家庭出现心理困扰。然而，封锁期间心理调整的变化尚未得到解决。本研究旨在调查父母压力水平的变化，儿童的行为困难，以及家庭被禁闭前和期间的亲子关系。此外，我们还考察了封闭期父母压力和儿童外化行为的预测因子。通过横断面调查，我们收集了841名意大利父母在家庭禁闭期间(4月20日‐ 5月18日)的数据。我们分析了在禁闭前和禁闭期间父母的压力水平，亲子关系和孩子的外化行为。父母压力(尤其是母亲)和儿童外化行为在封闭期有所增加。亲子关系保持稳定与否，可以预测父母压力，再加上身为母亲、孩子年龄更小、花在孩子身上的时间更少、远程工作的可能性更小。儿童外化行为的预测因素有: 男性、父母亲子时间较少、父母压力较大和儿童远程学习工作量较大。我们的研究结果表明，COVID‐19封锁对父母和儿童都有负面影响，研究表明积极的父母教养方式和对儿童的投入时间可能有助于减少流行病期间的限制对家庭调整的不利影响。

## BACKGROUND

In March 2020, the World Health Organization declared that the diffusion of COVID‐19—a respiratory disease caused by the novel virus SARS‐CoV‐2—had reached a pandemic level (WHO, 2020). Around that time, governments all over the world imposed different degrees of restrictions in order to prevent excessive infection spread and subsequent pressure on healthcare systems. Italy was among the first and most severely hit countries worldwide for the number of cases and deaths. On March 9, the Italian Government prescribed a strict lockdown—the *Phase 1*—that lasted until May 5, followed by very gradual resumptions of restrictions—the *Phase 2*. Schools were closed and did not reopen before the end of the school year in June.

About 9 millions of Italian students (from pre‐schools to high schools) were confined at home and about 8.8 millions of workers, among whom many parents had to start working remotely. Outdoor activities were banned or strictly limited as were after‐school sport or educational activities (Cluver et al., 2020). This implied a radical and abrupt change in family routines that could have had a serious influence on parents’ and children's psychological resources. In this regard, previous research highlighted the significant psychological impact of previous pandemics on parents and children. For instance, studies on Ebola revealed that the pandemic undermined the quality of parenting, increasing household conflict, and anxiety (Green et al., 2018). In addition, research on previous influenza A (H1N1) and severe acute respiratory syndrome (SARS) pandemics investigated the effect of disease‐containing measures. These studies showed that social distancing and quarantine may lead to adverse psychological consequences in a consistent percentage of families (Sprang & Silman, 2013). Therefore, it becomes essential to understand the psychological reactions of parents and children during the COVID‐19 lockdown, in order to identify and hence limit possible lockdown‐related challenges. Indeed, results in this direction could help to target support interventions for families that were particularly affected by the past lockdown experiences as well as to set up prevention strategies when restrictions have to be implemented again if new waves of the virus arise.

### The impact of COVID‐19 pandemic on families: adults and children's distress during lockdown

Recent studies on psychological adjustment during COVID‐19 pandemic in the general population highlighted increased mental health problems consisting of psychological stress, anxiety, and depression (Salari et al., 2020). These symptoms seem to be more severe in women (Liu et al., 2020; Moghanibashi‐Mansourieh, 2020; Wang, Di et al., 2020; Wang, Zhang et al., 2020), in younger adults (Ahmed et al., 2020; Huang & Zhao, 2020), and in highly educated individuals.

Although the majority of studies focused on the general population, COVID‐19 pandemic has generated new challenges and opportunities for families that may affect parental psychological distress and in turn children's well‐being (Cluver et al., 2020). Research on the impact of COVID‐19 pandemic on families is increasing, pointing out the importance of considering both individual risks as well as the interplay among parental and child factors (Di Giorgio et al., 2020; Donato et al., 2020; Morelli et al., 2020). Specifically, recent findings revealed that parents tend to report higher stress than non‐parents during the global COVID‐19 pandemic (Park et al., 2020; Russell et al., 2020). Furthermore, motherhood, individual psychological distress, and having younger children have been found to be predictive of higher parenting‐related exhaustion (Marchetti, Fontanesi, Mazza et al., 2020).

Besides parental burden, health agencies have warned against possible risks for children's physical and psychological well‐being as a consequence of long‐term home confinement (Aishworiya & Kang, 2021). Repercussions for children's mental health have been also empirically reported, with particular reference to emotional and behavioral problems (Di Giorgio et al., 2020; Moore et al., 2020). As in the context of other potential traumatic experiences, children's psychological outcomes during pandemic might be strongly dependent on their caregiver stress, resilience, and well‐being (Masten & Narayan, 2012; Russell et al., 2020). In this regard, psychological distress in parents has been found to affect children's emotional well‐being and hyperactivity/inattention (Marchetti, Fontanesi, Di Giandomenico et al., 2020; Morelli et al., 2020). In light of these findings, it is of particular relevance to further investigate the psychological impact of the COVID‐19 pandemic considering parental, couples, and children's distress in order to examine the process of family adjustment at different levels. Indeed, changes of the relations between those family factors during COVID‐19 outbreak, as well as how these relations might be modulated by COVID‐19 lockdown specific contingencies, remain poorly investigated yet.

### Parental stress during COVID‐19 pandemic and its relation with child externalizing behaviors

By taking care of their children and trying to meet their developing needs, parents provide and regulate children's exchanges with the environment that are crucial for their healthy growth and adaptation. However, parenting is a demanding job that may often represent a source of stress for mothers and fathers (Deater‐Deckard & Scarr, 1996). Parenting stress is a specific psychological reaction that can be defined as a negative or aversive response to parental obligations (Abidin, 1992; Holly et al., 2019; Wang, Di et al., 2020; Wang, Zhang et al., 2020). Moreover, parenting stress can be aroused when parenting demands are inconsistent with parent expectations and/or when parents have insufficient resources to meet the demands” (Holly et al., 2019). Thus, this adverse psychological response may have increased during home confinement, when external support was virtually unavailable and parents themselves were experiencing the difficult consequences of going through a pandemic. Recent studies realized during COVID‐19 outbreak showed that approximately one parents out of three perceived to be stressed to a moderate/high degree (Marchetti et al., 2020; Orgilés et al., 2020; Spinelli, Lionetti, Pastore et al., 2020). Notably, literature has widely shown that parenting stress constitutes a significant risk factor for child externalizing behaviors (Deater‐Deckard & Panneton, 2017), which is considered the most common reason for referral to child mental health services (Jones et al., 2017). The few existing timely studies suggest that also in the context of COVID‐19 home confinement, parenting stress might have detrimental effects on children, both at emotional and behavioral levels (Romero et al., 2020; Spinelli, Lionetti, Setti et al., 2020). Apart from these initial data, most of the studies focused on parents’ general psychological distress (e.g., depression, anxiety) and still little is known about the specific impact of COVID‐19 home confinement on parenting stress, its predictors, and its influence on child externalizing behaviors.

### Coparenting as a potential mediator of parental stress during COVID‐19 pandemic

Coparenting refers to the extent to which parents support or undermine each other's efforts in child rearing (Feinberg, 2003). A growing body of research suggests that coparenting may affect family well‐being, as it can directly or indirectly influence parenting dimensions and child adjustment (McHale et al., 2019). Previous studies highlighted that the level of coparenting alliance predicts parenting stress in non‐clinical mothers and fathers (Delvecchio et al., 2015). Moreover, coparenting has been found to be associated longitudinally with externalizing behaviors in children, from preschool to adolescence (Baril et al., 2007; Schoppe et al., 2001; Schoppe‐Sullivan et al., 2009). Interestingly, it has been suggested that the effect of coparenting is greater in situations where it is particularly important that parents work together, such as in crisis (Walsh, 2016). Nonetheless, coparenting continuity has been documented during periods of adjustment, such as the transition to parenthood (Altenburger et al., 2014). Moreover, a recent study on the psychological impact of COVID‐19 on Italian families of children with neurodevelopmental disorder highlighted continuity in coparenting before and during the lockdown and suggested the potential protective role of positive coparenting on children's externalizing behaviors (Omitted for blind review). However, evidence on the association of coparenting with parental stress and child behavioral difficulties during COVID‐19 home confinement are still scarce in families of typically developing children.

### COVID‐19‐related contextual factors that could influence parents’ and children's adjustment to home confinement

In addition to coparenting, other COVID‐19‐related contextual factors—pertaining family organization during home confinement—may have influenced parents and child well‐being during the pandemic. For instance, a recent study found that the lockdown has negatively affected the movement and play behaviors in children and youth (Moore et al., 2020), as they had limited access to outdoor spaces. Therefore, the possibility to use personal outdoor spaces within home might to some extent have reduced the impact of home confinement by allowing healthy motor and play activities. Also, parental work arrangement might have influenced their parental stress. Indeed, many parents had to work from home during the lockdown—balancing work and parenting tasks in the same environment—whereas others were required to work in presence. Initial findings suggest that changes in behavioral difficulties of children during lockdown were not related to parent working status (Di Giorgio et al., 2020). Yet, to the best of our knowledge, no studies have examined the impact of perceived feasibility of remote working on the parents’ stress. In a related way, the amount of time that parents could dedicate to shared activities with their children might have moderated parental and child distress. In fact, as a consequence of home confinement, the time spent by the parents with their children has massively increased in most families, but often parents had to carry out other activities while taking care of children. The parent involvement in child activities has recently been found to be associated with higher parenting stress during COVID‐19 home confinement (Spinelli, Lionetti, Setti et al., 2020). However, this result is in contrast with general findings linking parental time dedicated to children with parent‐child well‐being (Milkie et al., 2010; Townsend, 2002), therefore calling for further investigation.

Finally, during lockdown, school commitments have been transferred online implying challenges for both children and parents that might have impacted their psychological adjustment. A recent study showed that parents have experienced homeschooling as stressful and of poor quality (Thorell et al., 2021) during the first wave of COVID‐19. Moreover, Schmidt et al. (2020) also found that higher parental involvement in homeschooling during COVID‐19 outbreak was associated with lower quality of parent‐child interaction and lower positive affective responses in both parents and children. Despite increasing debate on parents’ and children's adjustment during lockdown, the contribution of COVID‐19‐related contextual factors has been poorly empirically investigated so far. Therefore, we aimed to explore the concurrent role of all those COVID‐19 lockdown‐related factors in predicting parental stress and child externalizing behaviors.

### The current study

Family psychological well‐being is characterized by parental, couple, and child factors. COVID‐19 lockdown produced changes and hardships in the families that might have impacted their psychological well‐being. On these premises, the study aimed to investigate psychological adjustment to COVID‐19 restrictions in Italian families considering parental stress, coparenting, and child externalizing behaviors, in order to capture the process of family adjustment during home confinement at different levels.

Specifically, the aim of the present study was twofold. Firstly, this study aimed at investigating changes between before and during COVID‐19 lockdown in perceived parental stress, child's externalizing behaviors, and coparenting. We hypothesized that perceived parental stress and child's externalizing behavior would increase during COVID‐19 home confinement, compared with the period before the lockdown. As for coparenting, no previous studies investigated changes in this dimension in the context of home confinement. However, based on the general literature, we expected coparenting continuity across time. Secondly, we aimed to examine which predictors contribute to parental stress and child externalizing behaviors during the lockdown, including coparenting itself, sociodemographic dimensions, and specific COVID‐related contextual variables (e.g., possibility to use outdoor spaces, current workplace, feasibility of remote working, child distance learning workload). With respect to parental stress, we hypothesized a negative predictive effect of motherhood, having younger children, and lower quality of coparenting relationship. As for COVID‐19‐related contextual variables, we expected that experiencing lower feasibility of remote working and higher child distance learning workload would increase perceived parental stress. Focusing on children, we hypothesized that higher distance learning workload would predict increased externalizing behaviors along with other variables that are more traditionally associated with behavioral difficulties, such as male gender, higher parental stress, and lower coparenting relationship. Finally, previous findings on the possible role of working status, time dedicated to the child, and use of outdoor spaces are either sparse or inconclusive. Thus, here we sought to test their predictive role on parents’ and children's stress with an explorative approach.

## METHOD

### Participants

The present study includes data from 602 parents (87% mothers) who completed an online survey on parenting in times of COVID‐19 pandemic during the period of strictest home confinement imposed by the Italian government (from March 9 to May 18). This sample was selected from a larger cross‐cultural project on parenting and child adjustment conducted in Italy, USA, and Spain during COVID‐19 pandemic crisis. Parents older than eighteen years old with at least one child age younger than 18 years old could participate in the survey. In total, 841 Italian parents completed the survey, among which, according to the present study purposes, we selected parents of children between 3 and 17 years old, without physical or intellectual disabilities as well as neuroatypical development. Moreover, since we estimated a completing time of 15 min on average, we excluded all the participants who completed the survey in less than 500 s to avoid the inclusion of casual or unreliable responses. The final sample consists of 602 participants. Characteristics of the study participants are described in Table 1.

**TABLE 1:** Characteristics of the study participants

Variables	Mother (*n* = 526)	Father (*n* = 76)	Statistics
Parent's age	40.58 (6.47)	41.96(6.43)	*p* = 0.08
Partner's age	44,65 (23.27)	39.77 (6.38)	*p* = 0.07
SES	38.01 (13.30)	40.05 (13.85)	*p* = 0.21
Having, or knowing someone, tested positive to the COVID‐19			
Yes	249 (46.3%)	34 (43.7%)	*p* = 0.38
No	277 (52.7%)	42 (55.3%)	
Number of children			
One	139 (26.4%)	29 (38.2%)	*p* = 0.07
Two	307 (58.4%)	40 (52.6%)	
More than two	80 (12.2%)	7 (9.2%)	
Current employment status			
Working	299 (56.3%)	61 (80.3%)	*p* < 0.01***
Not working	227 (40.2%)	15 (19.7%)	
Current working place			
Remote working	205 (69%)	31 (50.8%)	*p* < 0.01**
Workplace	92 (31%)	30 (49.2%)	
Children's gender			
Female	280 (52.2%)	37 (48.7%)	*p* = 0.26
Male	246 (46.8%)	39 (51.3%)	
Children's age	8.13 (3.76)	7.01 (3.13)	*p* = 0.01
Provenience			
North	419 (79.9%)	50 (66.6%)	*p* = 0.03*
Centre	31(5.9%)	9 (12.0%)	
South	74 (14.1%)	16 (21.3%)	

SES, socioeconomic status.

* *p* < 0.05.

** *p* < 0.001.

*** *p* < 0.001.

### Measures

#### Parental stress

Parental stress has been assessed by using 10 items from the *Parental Stress Scale* (PSS, Berry & Jones, 1995). We extracted a pool of items from the PSS to capture the demands and rewards of parenting related to the experience of home confinement during COVID‐19 outbreak. We selected 10 items in order to reduce the duration of the survey and minimize the drop‐out of participants. Specifically, we excluded the items regarding the general role of being a parent and personal development, and included items related to positive and negative themes of parenting, covering all the questionnaire areas (emotional benefits, self‐enrichment, personal development, demands on resources, opportunity costs, and restrictions). The scale describes feelings and perceptions about the experience of being a parent following a bidirectional perspective of parent‐child relationship. The participants were asked to indicate their agreement on a 5‐point Likert scale (1 = strongly disagree, 5 = strongly agree; range 10–50). The whole PSS has been widely used in parenting research (Berry & Jones, 1995) showing satisfactory psychometric properties(e.g., Berry & Jones, 1995, for a more recent review see Louie et al., 2017) also in clinical populations (Holly et al., 2019; Zelman & Ferro, 2018). In our sample, the shortened version revealed adequate internal consistency with Cronbach alpha values ranging from 0.757 to 0.814.

#### Coparenting

We used the brief form of the *Coparenting Relationship Scale* (CRS, Feinberg et al., 2012), a self‐reported questionnaire that includes 14 items focused on child‐rearing agreement, coparental support/undermining, division of labor, and joint management of family dynamics. Participants were asked to select the response that best describes the way they and their partner work together as parents, using a 0–6 Likert‐type scale (0 = Not true for us; 6 = Very true for us; range 14–84). According to previous studies, the CRS has shown good reliability, construct validity, and stability (Feinberg et al., 2012). Previous research on the 14‐item brief measure (CRS‐brief scale) has found good internal consistency both in mothers and in fathers, with Cronbach's alpha ranging from 0.81 to 0.89 (Feinberg et al., 2012). Furthermore, the CRS‐brief scale was found to be an excellent approximation of the full CRS scale, with a correlation of 0.97 for mothers and 0.94 for fathers (Feinberg et al., 2012) and the authors suggested that it can be used to adequately assess coparenting when time is limited. Subsequent studies have confirmed good internal consistency and that all subscales were adequately or strongly correlated with CRS‐brief total scores (Lamela et al., 2016; Lee et al., 2020). Cronbach's alpha on our sample, was 0.824 both during and before lockdown, showing good internal consistency.

#### Child's externalizing behaviors

We used 10 items selected from the Strengths and Difficulties Questionnaire (SDQ, Goodman, 2001), a widely used measure for the screening of child mental health problems. Specifically, we administered two subscales of the SDQ, namely the “hyperactivity/inattention” and the “conduct problems” scales, including five items each. The externalizing score ranges from 0 to 20, and it is the sum of both the conduct and the hyperactivity scales. For every item, participants were asked to rate to what extent the statement was true for their child, using a 3‐points response scale (0 = not true; 1 = somewhat true; 2 = completely true). Previous research has assessed good SDQ’s internal consistency, cross‐informant correlation, and test‐retest stability after 4–6 months (Goodman, 2001). Subsequent studies confirmed the good psychometric properties of the instrument, also for its Italian version (Di Riso et al., 2010; Tobia & Marzocchi, 2018) and cross‐culturally across European countries (Di Riso et al., 2010; Husky et al., 2020), with good screening detection especially for externalizing disorders. In our sample, Cronbach's alpha for SDQ externalizing scale ranges between 0.776 and 0.748 revealing good internal consistency.

#### Sociodemographic and contextual factors

Information about gender, age, socioeconomic status of the family, employment status (both in presence and remote working), number of children, having, or knowing someone, tested positive was collected. Furthermore, we asked to the parents to rate on a 5‐point Likert‐type scale other salient contextual factors, such as the possibility to use an outdoor spaces in their house, the time dedicated by the parent doing activities with the child, the feasibility of remote working in relation to the presence of children at home, and the child distance learning workload.

### Procedure

An anonymous online questionnaire was collected using a secure survey service software (Qualtrics) between the 20th of April and the 18th of May 2020. The population of interest was reached using a snowball sample recruitment strategy and by promoting the link on social media. The total duration of the survey was around 15 min. Each participant was allowed to fill out the questionnaire only once. The first pages of the online form described the aims and the methods of the study in detail. After reading the informed consent document and voluntarily accepting to participate, parents could start to complete the form. Participants could withdraw at any time by simply closing the webpage. All the collected data were anonymous and did not contain any personally identifiable information. The research project has been registered at the Department of (masked for review), following the ethical standards of the Italian Association of Psychology (AIP) and according to the European current regulation (European General Data Protection Regulation—GDPR UE 2016/67). For each item related to parental stress, coparenting relationship, and child externalizing problems, participants were asked to answer twice; they should refer to (i) the current situation during the lockdown period (T2); (ii) the last month before the COVID‐19 outbreak (T1), retrospectively. In case of multiple children, we asked the parent to refer to their youngest child.

### Analytic plan

Descriptive statistics were reported in Table 2. First, we performed Spearman bivariate correlations to examine potential covariates associated with parental stress and child externalizing score. Subsequently, we performed t‐test using parental gender, employment status, provenience, and having, or knowing someone who tested positive to COVID‐19 as independent factors and parental stress or child externalizing behaviors as dependent variables. Second, we conducted three separate repeated measures ANOVAs to test pre‐post differences on parental stress, coparenting relationship, and child externalizing problems, respectively, by using parent or child gender as between‐subjects factors. Next, we performed hierarchical multiple linear regression to examine whether demographic, COVID‐19‐related contextual variables, and parenting dimensions predicted parental stress (Table 3). The variables’ entrance was forced according to our research purposes. In the first block of the regression analysis, we entered parent gender and child age as predictors to control for variance related to these variables. In the second block of regression, we entered the contextual factors: use of outdoor spaces, employment status, and time dedicated by the parent with the child doing activities. Finally, in the third block, we entered the coparenting score. We replicated the same model in a subgroup of parents with children involved in homeschooling (*n* = 466), by adding child distance learning workload as predictor of parental stress in the fourth block. In addition, this regression model has been also replicated on a specific subsample of parents who work remotely (*n* = 236) replacing the variable “employment status” with “feasibility of remote working.” In fact, the subsample of 236 parents also responded to a specific question on feasibility of remote working related to the presence of the children at home rated on a 5‐point Likert‐type scale point. Furthermore, we conducted a hierarchical regression model to evaluate the contribution of demographic and contextual factors, as well as parenting dimensions (coparenting and parental stress), in predicting the child externalizing behaviors (Table 4). In the first block, we entered child age and gender as predictors; in the second block, we entered the use of outdoor spaces, and the time dedicated by the parent with the child doing activities; and in the third block, we entered coparenting relationship score and parental stress. Then, we replicated the same model in a subgroup of children who attended the distance learning (*n* = 466), by adding a fourth step that included the feasibility of child distance learning.

**TABLE 2:** Means, standard deviations, and statistics of the variables of interest (rows) by parent or child gender (columns)

Variables	Females	Males	Between statistics
Parental stress during COVID‐19	24.95 (7.46)	22.59 (6.57)	*F* = 5.09, *p* = 0.024
Parental stress pre‐COVID‐19	23.24 (6.02)	22.23 (5.46)	
Within statistics	*F* = 9.05, *p* = 0.003		
Coparenting during COVID‐19	73.86 (13.97)	76.95 (12.73)	*F* = 3.35, *p* = 0.068
Coparenting pre‐COVID‐19	74.00 (13.96)	77.26 (11.96)	
Within statistics	*F* = 0.417, *p* = 0.519		
Child ext. behaviors during COVID‐19	14.88 (3.46)	16.15 (3.79)	*F* = 21.8, *p* < 0.001
Child ext. behaviors pre‐COVID‐19	13.88 (2.96)	15.02 (3.28)	
Within statistics	*F* = 125.44, *p* < 0.001		

Abbreviation: Child Ext, Child externalizing behaviors.

**TABLE 3:** Hierarchical regression predicting parental stress during the lockdown

Variables	Parental stress					
	Model 1		Model 2		Model 3	
	*β*	CI	*β*	CI	*β*	CI
Parent gender	−0.115**	0.759, 4.33	0.124	0.97, 4.53	0.107**	−0.651, 4.08
Child age	−0.181***	−0.532, 0.205	−0.222	0.616, 0.287	−0.252***	−0.672, −0.354
Employment status			−0.062	−2.16, 0.311	−0.065	−2.16, 0.219
Outdoor spaces			−0.045	−0.994, 0.281	−0.029	−0.841, 0.388
Time with the child			−0.165***	−2.36, −0.773	−0.107*	−1.79, −0.235
Coparenting					−0.273***	−0.187, −0.103
Δ*R* ^2^	0.044	0.039	0.069			
*R* ^2^		0.082	0.151			

Note

Outdoor spaces: Possibility to use outdoor spaces during the lockdown; Time with the child: Amount of time spent by the parent doing activities with the child during the lockdown.

Abbreviation: CI, 95% confidence interval.

* *p* < 0.05.

** *p* < 0.01.

*** *p* < 0.001.

**TABLE 4:** Hierarchical regression predicting child externalizing behaviors during the lockdown

Variables	Externalizing behaviors					
	Model 1		Model 2		Model 3	
	*β*	CI	*β*	CI	*β*	CI
Child gender	−0.174**	−0.1.87, −0.686	−0.176***	−1.87, −0.713	−0.148**	−1.60, −0.562
Child age	−0.063	−0.146, 0.018	−0.103*	−0.187, −0.023	−0.024	−0.100, 0.052
Outdoor spaces			−0.050	−0.515, 0.120	−0.020	−0.397, 0.172
Time with the child			−0.196***	−1.31, −0.543	−0.105**	−0.855, −0.142
Coparenting					−0.071	−0.039, 0.002
Parental stress					−0.424***	0.174, −0.250
Δ*R* ^2^	0.036	0.043	0.069			
*R* ^2^		0.079	0.267			

Note

Outdoor spaces: Possibility to use outdoor spaces during the lockdown; Time with the child: Amount of time spent by the parent doing activities with the child during the lockdown.

Abbreviation: CI, 95% confidence interval.

* *p* < 0.05.

** *p* < 0.01.

*** *p* < 0.001.

## RESULTS

### Preliminary analyses

The correlational analyses revealed a significant negative association between parental stress and coparenting (*r* = −0.286, *p* < 0.001), child age (*r* = −0.141, *p* = 0.001), time dedicated doing activities with the child (*r* = −0.135), and feasibility of remote working (*r* = −0.486, *p* < 0.001). By contrast, socioeconomic status, the number of children, and the use of outdoor spaces were not correlated with parental stress. Focusing on group comparisons, the *t tests* and the one‐way ANOVAs yielded to non‐significant results for employment status and close contagions, whereas showed a significant effect of parent gender, with mother showing higher level of parental stress than fathers (*t*_(600)_ = −2.61, *p* = 0.009).

In addition, bivariate correlation showed significant negative association between child externalizing scores and parental stress (*r* = 0.479, *p* < 0.001), coparenting (*r* = −0.206, *p* < 0.001), time dedicated by the parent doing activities with the child (*r* = −0.159, *p* < 0.001), the use of outdoor spaces (*r* = −0.092, *p* < 0.05), and the feasibility of child distance learning (*r* = 0.126, *p* < 0.01). The correlation between child externalizing problems and child age, socioeconomic status, number of children were non‐significant. Finally, a significant correlation between child and parent age emerged (*r* = −0.621, *p* > 0.001).

### Differences pre‐post lockdown (repeated measures ANOVAs)

Results on parental stress revealed significant effects of parent gender (*F*_(1,600)_ = 5.09, *p* = 0.02), time (*F*_(1,600)_ = 9.00, *p* < 0.01), and their interaction (*F*_(1,600)_ = 3.86, *p* = 0.05), with higher increased of parental stress in mothers than in fathers. No significant differences emerged with regard to coparenting relationship.

Results on child externalizing behaviors indicated significant main effects of time (*F*_(1,600)_ = 125.44, *p* < 0.001), with increased symptoms during vs before the lockdown period, and a main effect of gender (*F*_(1,600)_ = 21.82, *p* < 0.001), with males showing higher scores than females (*F*_(1,600)_ = 21.81, *p* < 0.001).

### Hierarchical regression on parental stress (total sample *n* = 602)

In line with our second hypotheses, hierarchical regression analyses were performed to investigate the contribution of specifically selected predictors on parental stress (Table 3). The overall regression model explains around 15% of the variance. In the first step, parent gender and child age significantly contributed to the understanding of parental stress explaining only 4% of the variance. In the second step, only the time dedicated by the parent doing activities with the child was statistically significant (*β* = −0.165, *p* < 0.001). In the final model, there was a significant predictive effect of parent gender (*β* = 0.107, *p* = 0.007), child age (*β* = −0.252, *p* < 0.001), time dedicated by the parent doing activities with the child (*β* = −0.107 *p* = 0.011), and quality of coparenting relationship (*β* = −0.273 *p* < 0.001). Each step adds a significant contribution to the overall model (all *p* < 0.05).

We also tested the contribution of perceived child distance learning workload on parental stress replicating the model on a subgroup of parents whose children were involved in distance learning (*n* = 466), which emerged as a significant predictor of parental stress during lockdown (β = 0.150, *p* = 0.001).

### Hierarchical regression on parental stress, subgroup of parents who worked remotely (*n* = 236)

Next, we replicated this model on a subsample of parents who worked remotely (*n* = 236) by replacing employment status with the feasibility of remote working in the second block of the model. This hierarchical regression analysis revealed that the predictors explain around 25% of the variance. In the final model, feasibility of remote working (*β* = −0.417 *p* < 0.001) and quality of coparenting (*β* = −0.232 *p* < 0.001) contributed significantly to the model, while parent gender, child age, possibility to use outdoor spaces, and time dedicated by the parent doing activities with the child did not show a significant predictive effect on parental stress. In this case, the second step of analysis added a significant amount of variance to the model (18.3%).

### Hierarchical regression on child externalizing behaviors, total sample (*n* = 602)

Furthermore, hierarchical regression analyses were performed on child externalizing problems as reported by the parent. (Table 4). Firstly, we performed a hierarchical regression considering all the participants, and secondly, we replicated this model on the subgroup of children who followed distance learning. The overall model explained 26% of the variance. In the first step, child gender, but not child age, significantly contributed to the understanding of externalizing problems, with males showing higher scores than females (*β* = −0.174, *p* < 0.001). In the second step, the time dedicated by the parent doing activities with the child was significant (*β* = −0.195, *p* < 0.001), while the possibility to use outdoor spaces did not account for a significant amount of variability. In the final step, there was a significant predictive effect of child gender (β = 0.148, *p* < 0.001), time dedicated by the parent doing activities with the child (*β* = −0.105 *p* = 0.006), and parental stress (*β* = 0.424 *p* < 0.001). Coparenting relationship only showed a marginal trend toward significance (*β* = −0.071, *p* < 0.070). The third block of the regression adds a significant proportion of the variance (18.6%).

### Hierarchical regression on child externalizing behaviors, subgroup of children who did distance learning (*n* = 466)

Next, we replicated this model on a specific subsample of children who did distance learning by entering the feasibility of child distance learning as an additional predictor in the last block. This hierarchical regression analysis revealed that the predictors explain around 26% of the variance. In the final model, child gender (*β* = −0.156, *p* < 0.001), time dedicated by the parent doing activities with the child (*β* = −0.087, *p* = 0.046), parental stress (*β* = 0.405 *p* < 0.001), and feasibility of child distance learning (*β* = −0.108, *p* < 0.011) contributed significantly to the model. Unlike the child age, the possibility to use outdoor spaces and coparenting did not show a significant predictive effect on externalizing behaviors.

## DISCUSSION

Evidence of psychological distress in families during COVID‐19 home confinement are arising. However, the perceived changes in psychological adjustment during home confinement with respect to the period before the pandemic have not been addressed yet. Furthermore, little is known about the role of coparenting and other specific COVID‐19 contextual variables on parental stress and children's behavioral difficulties in the Italian context.

To fill this gap, the present study investigated changes in the perception of parental stress, children's behavioral difficulties, and coparenting before and during the lockdown period. Moreover, in addition to specific factors that are traditionally associated with parental stress and children's externalizing behaviors, we examined the contribution of specific COVID‐19‐related contextual variables that could influence parents’ and children's adjustment to home confinement.

### Parental stress increase and predictors during home confinement

Firstly, our findings confirmed the detrimental effect of pandemic restrictions on family adjustment, showing an increase of psychological distress in both parents and children. We found that mothers reported higher levels of parental stress than fathers both prior and during the lockdown, consistently with previous studies showing more severe symptoms of psychological distress in women (Huang & Zhao, 2020) also in the Italian context (Di Giorgio et al., 2020). Moreover, although the demands of COVID‐19 pandemic were associated with increased scores in both parents, we found that the mothers reported a higher increase of parental stress during lockdown. This suggests that the impact of home confinement may place a much higher burden on mothers as they are often the primary caregivers. In this regard, as a result of the stressful conditions related to the pandemic, mothers may tend to perceive increased demands of daily parenting and less available personal resources, considering their child more difficult to care for. Moreover, in the Italian context, mothers may be more exposed to the risks of COVID‐19 given the higher prevalence of women in the sanitary system and the nursing homes, the greater risk to lose their job, as well their higher involvement in homeschooling and childcare (Istat, 2020). We also found that parental stress during the COVID‐19 was explained by child age, with parents of younger children reporting to experience greater difficulties, in agreement with prior research on Italian parents’ distress during the pandemic (Di Giorgio et al., 2020). Consistently with a bidirectional perspective, parents may perceive as more stressful and demanding the developmental needs of younger children, who are exclusively dependent on their caregivers during lockdown and require continuative supervision and greater parental involvement. Our findings also highlighted that the time dedicated by the parent in doing activities with the child and the quality of coparenting relationship contributed to significantly decrease the level of parental stress during COVID‐19 lockdown. According to Spinelli, Lionetti, Setti et al. (2020), the more stressed parents may be less involved in activities with their child. Thus, it is plausible that not being able to spend time in doing activities with the child, especially in such stressful conditions, may increase the imbalance perceived by the parents between the child requests and the abilities to deal adequately with them. A recent Italian study has also highlighted that mothers had a distorted time experience during the quarantine (Di Giorgio et al., 2020), which may reduce the possibility to organize structured and planned activities with the child. Another novel and interesting result that emerged from our study is the effect of coparenting as predictors of parental stress during COVID‐19 lockdown. This result confirms the influence of couple‐related factors on parental stress during stressful circumstances (Donato et al., 2020). Additionally, we extended previous findings that were limited to Italian children with neurodevelopmental disorder (Omitted for blind review), showing that quality of coparenting influences parental stress during pandemic also in families of children with typical development. With respect to COVID‐19‐related contextual factors, feasibility of remote working emerged as a key factor in predicting parenting distress. Specifically, our findings showed the detrimental effect of remote working on parental stress, consistently with previous studies (Chung et al., 2020; Romero et al., 2020; Spinelli, Lionetti, Pastore et al., 2020). The work‐family balance may constitute a conspicuous challenge for parents during a time of pandemic that may hamper parental stress, which in turn can affect parenting cognitions and practices. In addition, our results highlighted the negative influence of distance learning workload on parental stress, extending previous findings on the effect of parental involvement in homeschooling on the quality of parent‐child interaction (Schmidt et al., 2020). Therefore, the burden of homeschooling workload during the pandemic seems to have an impact not only on children, but also on parents.

Finally, we found that the level of parental stress did not change according to family SES, provenience and COVID‐19 contagion in relatives/friends, current employment status, and the possibility of using outdoor spaces. In particular, since the vast majority of our sample reported a medium SES, the poor variability in our sample may have hidden the effect of SES on parenting dimensions. With respect to COVID‐19 infection, no information on the degree of illness severity has been collected; thus, this limitation may explain the lack of associations between experience of contagion and parental distress.

### Child externalizing behaviors increase and predictors during home confinement

With respect to children's outcomes, our results showed a significant increase of externalizing behaviors during lockdown. We found higher levels in males than females at both the timepoints, consistently with prior research documenting the higher occurrence of externalizing behaviors in males during childhood and adolescence (Bongers et al., 2004; Moffitt et al., 2008). Nevertheless, our results showed that males and females exhibit a similar increase of externalizing behaviors across time, suggesting that the impact of the lockdown is not dependent on gender. This result confirms previous findings on the psychological impact of COVID‐19 on children's behavioral adjustment as perceived by the parents (Di Giorgio et al., 2020; Orgilès et al., 2020). Focusing on the predictors of children's externalizing behavior during home confinement, we confirmed the role of sociodemographic factors, such as child age and gender. Specifically, we found that higher increase of externalizing behaviors was predicted by younger age and being a male. These results are in agreement with previous research, highlighting that younger children are more likely to manifest externalizing behaviors, which tend to decrease across development (Shaw et al., 2005). Preschoolers may be more vulnerable during quarantine than older children, as they have less psychological resources to cope with stressful circumstances (Franks, 2011), less opportunities to keep contacts with other people during quarantine using electronic devices (Romero et al., 2020), and rely almost exclusively on their caregivers as relational sources. Moreover, In Italy, homeschooling was activated only for adolescents during the first wave of pandemic, while for the younger children the school activities and the contact with teachers and schoolmates were just stopped. Thus, the school closure and the lack of structured educational activities may have had a greater impact on younger children and their behavioral functioning. Our findings also highlighted that children were perceived as showing higher levels of hyperactivity and conduct problems when parents reported to be less involved in activities with them. These results extend previous studies outside the context of pandemic, which revealed that children who experience less positive parental involvement and unsupervised time showed higher externalizing behaviors across childhood (Beyer et al., 2003). This is of particular importance during the period of home confinement in view of the greater opportunities that parents and children may have to share time doing activities together.

In our sample, the stronger predictor of child externalizing behaviors was parental stress. The link between parental stress and child adjustment is largely supported by prior research (Neece et al., 2012), including the recent studies on the impact of COVID‐19 outbreak on parenting (Romero et al., 2020; Spinelli, Lionetti, Setti et al., 2020). However, it is important to note that parents’ perception of their children's outcomes may be influenced by higher parental distress (Briggs‐Gowan et al., 1996). Thus, parents may overreport their child behavioral difficulties as a consequence of the caregiver burden experienced during the quarantine period (Russell et al., 2020). In general, we confirmed the crucial role of these well‐established predictors of child behavioral difficulties also in the context of stressful circumstances, as is the case of COVID‐19 pandemic.

Furthermore, beyond typical findings on family processes, we found that the child distance learning workload (as perceived by the parents) significantly predict child behavioral difficulties, extending previous findings about the potential negative effects of homeschooling on children's experience (Ishimoto et al., 2020; Thorell et al., 2021). This could be related to the difficulty of learning from new digitally mediated practice, as well as to the decreased support received from the teachers and the schoolmates. Unplanned school closure may also have increased feelings of social isolation, which has been found to be linked to significant changes in child behaviors and mental health difficulties (Loades et al., 2020; Tichovolsky et al., 2013), particularly in younger children. As is the case of parental stress, child externalizing behaviors were not influenced by family SES and the possibility of using outdoor spaces. Similarly to parental stress, this may be due to the reduced variability of these factors in our participants.

### The role of coparenting relationship

We did not find significant differences before and during the lockdown on quality of coparenting in both mothers and fathers, consistently with prior research showing its continuity across time (McHale et al., 2004). Nevertheless, our findings highlighted the importance of good reciprocal support and agreement between the parents in preventing parental stress also during stressful conditions such as the home confinement. Given the high level of energy required to parents to deal with cumulative stressors and multiple tasks during COVID‐19 lockdown, the involvement of multiple caregivers may be even more helpful than in non‐emergency time for effectively managing the daily challenges (Walsh, 2016).

Although the predictive effect of coparenting on externalizing behaviors was not significant, our findings suggest the presence of an indirect relationship via the parenting stress. Indeed, coparenting was a significant predictor of parental stress that, in turn, significantly predicted child externalizing behaviors. This result is in line with previous findings underlining the key role of coparenting relationship and family processes on child behaviors (Schoppe et al., 2001). To our knowledge, this is the first study confirming the direct and indirect role of the quality of coparenting relationship during pandemic in families of children and adolescents with typical development.

## CONCLUSIONS AND LIMITATIONS

The current study highlighted the impact of COVID‐19 outbreak on psychological distress of parents, particularly in mothers, and younger children. Coparenting emerged as a crucial factor that mitigate parental stress and, in turn, parental perception of child behaviors. Parental stress and the time dedicated by the parent doing activities with the child significantly contribute to child externalizing behaviors. As regard to COVID‐19 contextual factors related to home confinement, we found a significant predictive effect of feasibility of remote working and distance learning workload on parental stress and children's behavioral difficulties. These findings confirmed the relevance of family processes for parents’ and children's health according to a bidirectional perspective, also during pandemic.

In sum, this study confirms and extends some of initial findings on family adjustment during COVID‐19 pandemic and brings some novel evidence on the influence of specific COVID‐19‐related factors on parental, couple, and child well‐being during lockdown. However, some limitations need to be acknowledged. Firstly, we used a cross‐sectional design to assess changes across time; thus, another possible explanation of the difference in parental stress and child externalizing behaviors between pre‐lockdown and during lockdown could rely on the fact that the strains during the lockdown period might have positively biased the parental memories of the period before the pandemic. Second, the sample size was imbalanced, with a greater number of mothers compared to fathers. In this regard, as highlighted by previous studies (Seidler et al., 2016), men may have more difficulties in recognizing and communicating their psychological distress due to masculine norms. This imposes caution in interpreting the parental gender effect. Third, the brevity and the technique of the data collection should also be acknowledged as a potential drawback of the study, since participation in the study may be limited to parents who own digital devices and are familiar with social media. This may imply a greater presence of medium‐high compared to lower socioeconomic class in our sample. Hence, future research focusing on the effect of COVID‐19 lockdown on lower‐income families is desirable. Fourth, child externalizing behaviors were only reported by the parent without considering other child informants (i.e., teachers). Furthermore, the absence of a follow‐up data after the lockdown period represents a limitation to the understanding of the middle‐term impact of quarantine on parents and their children. Measures of parenting and child dimensions related to the months before the lockdown may be subjected to recall bias, since the stressful situation of the current pandemic could influence participants’ reflections to the past. In this regard, we used static measures of adult and child distress during the pandemic as dependent variables—rather than the indices of change—because participants that reported higher levels of stress or behavioral difficulties also before lockdown may not show significant variations across time, as they did already reach the ceiling score in the first time point. Future studies should focus on predictors of changes in parenting and child dimensions using a longitudinal approach. Despite the described limitations, the present study offers some insights for healthcare interventions and policies in time of pandemic. Firstly, it is essential to consider the complex interplay between dyadic and individual parenting variables to understand the determinants of family psychological well‐being child behaviors also in stressful situations, such as COVID‐19 outbreak. Healthcare practitioners should consider that parental support is essential during a pandemic, in order to prevent mental health difficulties in mothers, fathers, and children given the multiple interrelationships that occur within the family context. Being confined at home did not directly imply to share more dedicated time with their children for doing activities, that may be hampered by homeschooling and homeworking burden. Thus, helping parents to capitalize the opportunity to spend more time doing activities with their children should be considered an important protective factor during lockdown. Furthermore, particular attention needs to be paid by policymakers to the burden experienced by mothers and younger children, and to the difficulties reported by parents in combining remote working and childcare, including homeschooling. Notably, both parents and children may benefit from more proportionate distance learning workload during the pandemic. Finally, since coparenting plays a key role for parenting stress also during pandemic, policies should facilitate parental collaboration in order to contain the psychological impact of home confinement.

In conclusion, results of our study contribute to understand the impact of lockdown on family psychological adjustment. This is of particular relevance to target support interventions for families that were more affected by the past lockdown experience and to set up prevention strategies in case of new contagion waves of COVID‐19, as well as of other future pandemics.

## CONFLICT OF INTERESTS

None.

## ETHICAL APPROVAL

The authors assert that all procedures contributing to this work comply with the ethical standards of the relevant national and institutional committees on human experimentation and with the Helsinki Declaration of 1975, as revised in 2008.
